# Serum uric acid to creatinine ratio and metabolic syndrome in postmenopausal Chinese women

**DOI:** 10.1097/MD.0000000000019959

**Published:** 2020-04-24

**Authors:** Jing Tao, Xin Shen, Jie Li, Erdenbat Cha, Pei-Pei Gu, Jun Liu, Wei Zhu, Lin-Long He, Guo-Qing Li, Zhao Wang

**Affiliations:** Department of Cardiology, People's Hospital of Xinjiang Uygur Autonomous Region, Urumqi, PR China.

**Keywords:** creatinine, metabolic syndrome, postmenopausal, uric acid

## Abstract

This study aimed to investigate the association between Serum Uric Acid (UA) to Creatinine (Cr) Ratio (UA/Cr) and metabolic syndrome (MetS) in postmenopausal women.

A total of 455 patients with MetS and 457 age- and gender- matched controls were included in the present retrospective study. Serum levels of total cholesterol (TC), triglycerides (TG), high-density lipoprotein cholesterol (HDL-C), low-density lipoprotein cholesterol (LDL-C), fasting plasma glucose (FPG), Cr, and UA were measured. We employed logistic regression analysis to investigate the association between serum UA/Cr and MetS in postmenopausal women.

Serum UA/Cr levels were significantly higher in patients with MetS than that in control subjects (*P* < .05). In the correlation analysis, serum UA/Cr showed a significantly positive correlation with age, hypertension, systolic pressure (SBP), diastolic pressure (DBP), Waist, body mass index (BMI), TG, UA and negative correlation with type 2 diabetes mellitus (T2DM) and Cr (*P* all < .001). Moreover, multivariate analysis revealed that serum UA/Cr was still an independent risk factor for MetS (OR = 2.928, 95% CI = 2.385–3.596, *P* < .001) after adjustments for other confounders.

Serum UA/Cr are strongly associated with the risk of MetS in postmenopausal Chinese women.

## 1 Introduction

Metabolic syndrome (MetS) is a cluster of metabolic disorders including abdominal obesity, raised blood pressure, high triglycerides (TG), low high-density lipoprotein cholesterol (HDL-C) and hyperglycemia.^[1]^ As a major worldwide public health problem, the prevalence of MetS has grown markedly over the past decades.^[2–4]^ In China, the age-standardized prevalence of MetS was 9.8% in men and 17.8% in women in 2001, and the figures have increased to 31.0% in men and 36.8% in women in 2010.^[5]^ It has been demonstrated that MetS is associated with the increasing mortality of type 2 diabetes mellitus (T2DM), cardiovascular disease and various other causes.^[6–9]^ Postmenopausal women have an increased risk of MetS and epidemiological studies showed that prevalence of MetS is significantly higher in postmenopausal women too.^[10–13]^

Uric Acid (UA) is the final product of purine metabolism and is mainly eliminated in the urine.^[14]^ Serum uric acid (SUA) level does represent an important risk factor for cardiovascular disease.^[15]^ It has been demonstrated that SUA level is closely related to conditions such as obesity, endothelial dysfunction, oxidative metabolism, impaired glucose metabolism, platelet adhesiveness and hypertension.^[16–18]^ In the First National Health and Nutrition Examination Survey (NHANES I) study in USA, a total of 5926 subjects were enrolled to investigate the association of SUA level with cardiovascular mortality and the result showed that increased SUA levels are independently and significantly associated with risk of cardiovascular mortality in an average of 16.4 years of follow-up.^[19]^ Franse et al performed a cohort study in a randomized trial in 4327 patients with isolated systolic hypertension and found that SUA independently predicts cardiovascular events in older persons with isolated systolic hypertension.^[20]^ Furthermore, in the community-based population of the Cardiovascular Health Study performed by Ekundayo et al, SUA level was associated with an increased risk of incident heart failure and each 1 mg/dL increasing in SUA level was associated with a 12% increasing in incident heart failure (HR = 1.12, *P* = .006).^[21]^ Serum creatinine (Cr) is a commonly used indicator of kidney decline. Evidences have demonstrated that Serum Cr was associated with the increased risk of T2DM, hypertension, obesity and CAD.^[22]^ Recently, renal function-normalized SUA (UA/Cr) has appeared as a new biomarker and is considered to reflect endogenous UA levels more precisely than SUA level. Several studies have suggested that serum UA/Cr ratio was significantly associated with chronic obstructive pulmonary disease, chronic kidney disease, β-Cell function in type 2 diabetes mellitus patients.^[23–25]^ However, there are limited studies focused on the relationship between serum UA/Cr ratio and MetS. The aim of the present study was to investigate the association between serum UA/Cr ratio and MetS in Chinese postmenopausal women.

## 2 Methods

### 2.1 Study subjects

All patients with MetS and control subjects were recruited from the People's Hospital of Xinjiang Uygur Autonomous Region from 2016 to 2018. A total of 455 patients with MetS and 457 age- and gender- matched controls were included in the present retrospective study. MetS was diagnosed according to the National Cholesterol Education Program Adult Treatment Panel III (NCEP-ATP III) criteria.^[26]^ In detail, the definition of MetS requires the presence of any 3 or more of the following 5 abnormalities:

1. elevated waist circumference: ≥90 cm in men or ≥80 cm in women;
2. systolic blood pressure ≥130 mm Hg or diastolic blood pressure ≥85 mm Hg or established treatment of already diagnosed hypertension;
3. elevated TG: ≥1.7 mmol/L or on drug treatment for elevated TG;
4. reduced HDL-C: < 1.03 mmol/L in men or < 1.3 mmol/L in women or on drug treatment for reduced HDL-C; and
5. abnormal fasting plasma glucose: ≥5.6 mmol/L or on drug treatment for elevated glucose. This study was approved by the Ethics Committee of the People's Hospital of Xinjiang Uygur Autonomous Region (Urumqi, China).

### 2.2 Risk factors and definitions

In order to get a complete medical history, information on anthropometric measurements and blood biochemical examination were performed in all participants. Data on age, gender, systolic blood pressure, diastolic blood pressure, Waist, body mass index (BMI), smoking status, drinking status, T2DM, Serum concentrations of total cholesterol (TC), TG, HDL-C, low-density lipoprotein cholesterol (LDL-C), fasting plasma glucose (FPG), Cr and UA were collected.

Hypertension was defined as a systolic blood pressure of ≥140 mmHg and/or a diastolic blood pressure of ≥90 mmHg in at least 2 measurements or use of any antihypertensive drug. Diabetes mellitus was defined as 2 FPG level ≥ 7.0 mmol/l or a prior diabetes diagnosis and/or using a diabetes drug. Smoking was defined as current smoking. Drinking was defined as alcohol consumption ≥2 times per week. BMI was calculated as the weight in kilograms divided by the square of the height in meters (kg/m^2^).

### 2.3 Laboratory analysis

Peripheral venous blood samples of the patients were obtained for the assessment of routine laboratory parameters after 12-hour fasting. Laboratory tests were performed at the core laboratory of the People's Hospital of Xinjiang Uygur Autonomous Region. TC, TG, HDL-C, LDL-C, uric acid, and creatinine levels were directly measured using the homogeneous enzymatic colorimetric assay (Roche Diagnostics) on an automatic analyzer (Cobas 8000).

### 2.4 Statistical analysis

All statistical analyses were performed with the Statistical Package for the Social Sciences (SPSS) 17.0 software package (SPSS Inc, Chicago, IL). Continuous variables were presented as the mean ± standard deviation (SD) and were compared using an independent samples *t* test. Categorical variables were expressed as numbers and percentages and were analyzed using chi-square tests. Relationships between serum UA/Cr and other variables were tested by Pearson correlation analysis. Logistic regression analyses were used to investigate potential factors associated with MetS and components of MetS. All statistical tests were 2-sided, and statistical significance was determined at *P* < .05.

## 3 Results

### 3.1 Clinical characteristics of the study population

**Figure 1:**
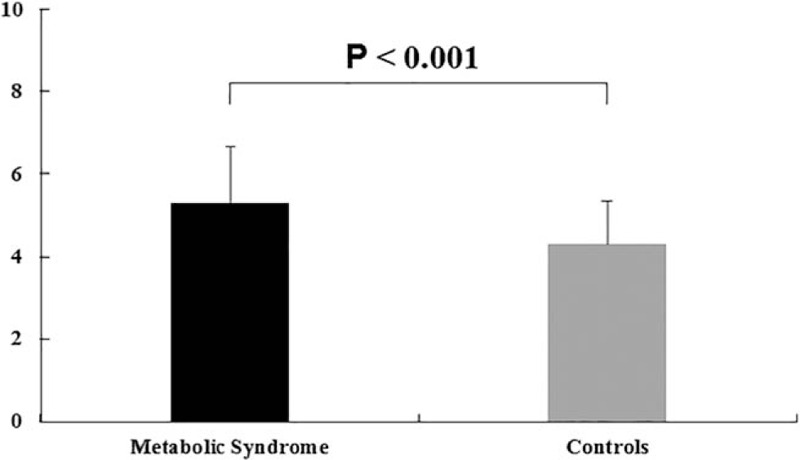
UA/Cr ratio was significantly higher in patients with metabolic syndrome than controls.

A total of 912 subjects were recruited in the present study. The mean age of the study population was 62.41 ± 7.58. All participants were divided into MetS and control group according to the presence or absence of MetS. The baseline clinical characteristics of 455 MetS patients and 457 control subjects were shown in Table 1. BMI, Waist, FPG, TG, TC, UA and prevalence of hypertension and T2DM were more likely to be higher in patients with MetS than that in control subjects (*P* all < .05). Serum UA/Cr were also more likely to be higher in patients with MetS than that in control subjects (*P* < .001) (Fig. 1). While, HDL-C, LDL-C and SCr were more likely to be lower in patients with MetS than that in control subjects (*P* all < .05).

**Table 1:**
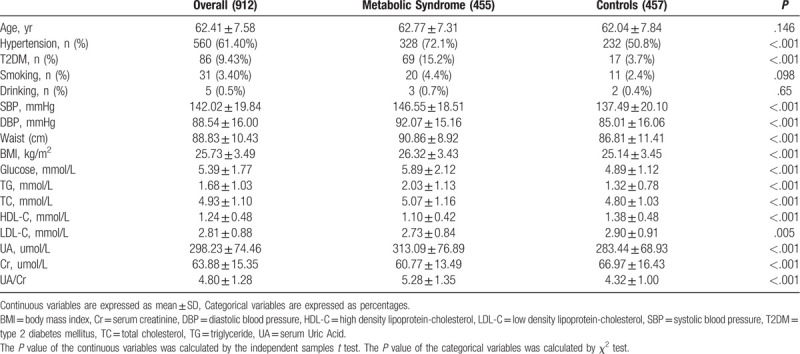
Baseline clinical characteristics of the study population.

### 3.2 Correlations between serum UA/Cr and other variables

In the correlation analysis, serum UA/Cr showed a significantly positive correlation with age (r = 0.189, *P* < .001), hypertension (r = 0.247, *P* < .001), SBP (r = 0.273, *P* < .001), DBP (r = 0.225, *P* < .001), Waist (r = 0.310, *P* < .001), BMI (r = 0.271, *P* < .001), TG (r = 0.065, *P* = .049), UA (r = 0.647, *P* < .001). In addition, serum UA/Cr also showed a significantly negative correlation with Diabetes (r = −0.075, *P* = .023), serum Cr (r = −0.430, *P* < .001) (Table 2).

**Table 2:**
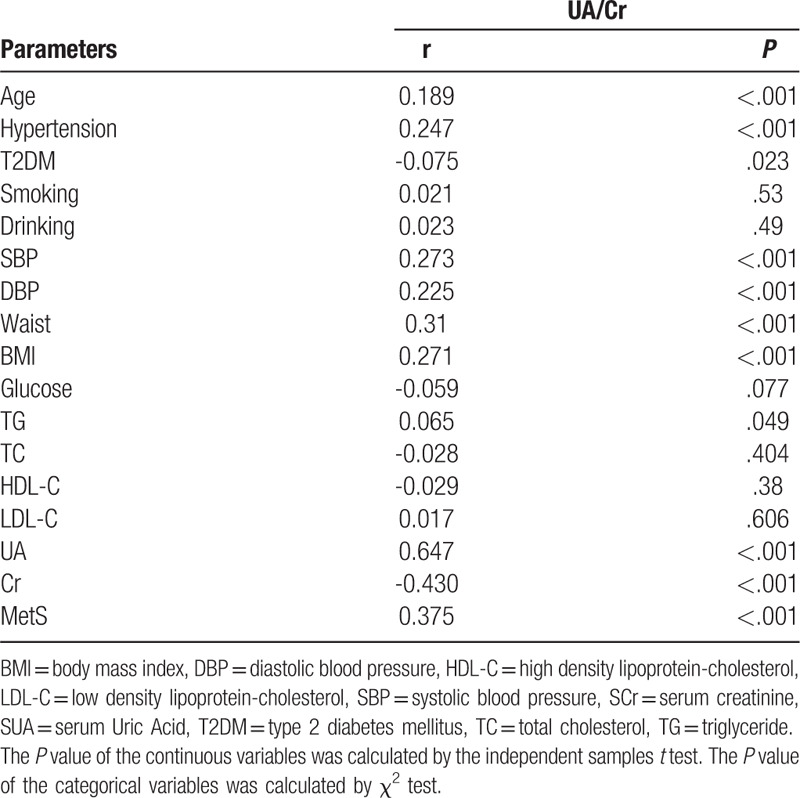
Correlations between serum UA/Cr and other variables.

### 3.3 Multivariable logistic regression analyses of the major confounding factors for MetS

After the multivariate adjustments for the confounders, such as age, hypertension, smoking, drinking, BMI, waist, glucose, TG, TC, HDL-C and LDL-C, serum UA/Cr was still an independent risk factor for MetS (OR = 2.928, 95% CI = 2.385–3.596, *P* < .001) (Table 3).

**Table 3:**
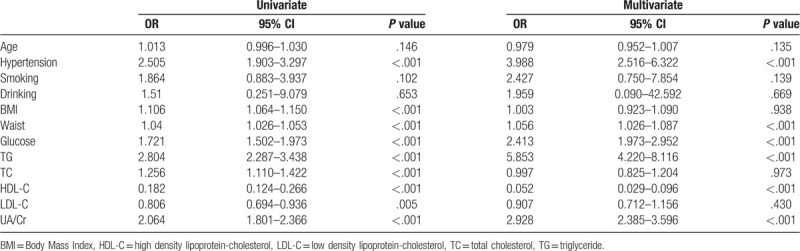
Logistic Regression Analysis for Metabolic Syndrome.

### 3.4 Multivariable logistic regression analyses of the major confounding factors for the components of MetS

The multinomial variable was recoded into 5 categories: Central obesity, Hypertriglyceridemia, Low HDL-Cholesterol, Hypertension, and Hyper-glucose. The independent variables were age, hypertension, smoking, drinking, BMI, waist, glucose, TG, TC, HDL-C, LDL-C, and serum UA/Cr. After the multivariate adjustments for the confounders, UA/Cr was still an independent risk factor for central obesity and hypertension (OR = 2.122, 95% CI = 1.687–2.669, *P* < .001; OR = 1.899, 95% CI = 1.570–2.296, *P* < .001) (Table 4).

**Table 4:**
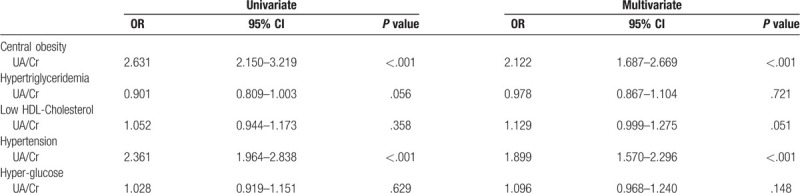
Logistic regression analysis for the components of metabolic syndrome.

## 4 Discussion

In the present study, we investigated associations between serum UA/Cr ratio and MetS in postmenopausal women. Our results showed that serum UA/Cr ratio was higher in patients with MetS when compared with controls. There was a positive association between the increase in serum UA/Cr ratio and the prevalence of MetS. In addition, our results also showed that serum UA/Cr ratio was independently associated with MetS susceptibility even after adjustment for other baseline parameters. To the best of our knowledge, this is the first study to demonstrate the association of serum UA/Cr ratio and MetS in postmenopausal women.

Postmenopausal status is associated with an increased incidence of MetS. Epidemiological and clinical studies have shown that MetS is more relevant to postmenopausal women than premenopausal ones. Ricardo et al performed a cross-sectional study in pre- and postmenopausal women and found that the prevalence of MetS was 9.4% in premenopausal women and 22.2% in postmenopausal women.^[27]^ Data from the Third National Health and Nutrition Examination Survey suggested that MetS was an independent risk factor for all-cause mortality, cardiovascular mortality, cardiac mortality and non-cardiovascular mortality in the subgroup of postmenopausal women. At the same time, the hazard ratio was also stronger in postmenopausal women and even became nonsignificant in the premenopausal women.^[28]^ Hence, it is important to identify high-risk individuals for MetS in Chinese postmenopausal women.

The association between SUA and MetS has long been recognized. In a meta analyses involving 54,970 participants and 8719 MS cases, elevated SUA levels led to an increased risk of MetS and were consistent with a linear dose-response relationship.^[29]^ Yadav et al performed a population-based cohort study in 1590 healthy adults aged 40 to 70 years, and found that SUA may predicted as a risk factor for developing MetS during a mean of 2.6 years of follow-up.^[30]^ Yang et al performed a prospective study of 3857 subjects who were free of MetS and found that hyperuricemia was an independent risk factor for MetS in women during a mean follow-up of 5.41 years.^[31]^ Uric acid (UA) is the final oxidation product of purine metabolism in humans and SUA level is influenced by kidney function. However, most previous studies ignored the contribution of kidney on UA levels. Serum UA/Cr, a function-normalized SUA index, is considered to be better representative of endogenous serum uric acid and may be better correlated with metabolic diseases. In the present study, we found that serum UA/Cr was significantly higher in MetS patients than that in controls. After the multivariate adjustments for the confounders, serum UA/Cr was still an independent risk factor for MetS. The following description may explain the mechanisms that link SUA to MetS. First, Hyperuricemia may lead to the impaired of endothelial function and decreased release of nitric oxide from endothelial cells. While, the uptake of glucose in skeletal muscle is partly depended on the increasing blood flow which are mediated by the release of nitric oxide from endothelial cells. Therefore, UA may exacerbate insulin resistance by inhibiting the bioavailability of nitric oxide.^[32–33]^ The second mechanism may be the inflammation and oxidative stress caused by SUA. Previous studies have demonstrated that the oxidative changes induced by hyperuricemia in adipocytes is a key in causing the metabolic syndrome in obese mice.^[34]^

The association between SUA and all MetS components have been studied in other studies. In the present study, we found that serum UA/Cr was still an independent risk factor for central obesity and hypertension after the multivariate adjustments for the confounders. The mechanism is still unclear, but insulin resistance induced by hyperuricemia is considered to be associated with the development of these metabolic disorders. In addition, we also found that serum UA/Cr showed a significantly negative correlation with T2DM. The reason may be that long-term chronic hyperglycemia in patients with T2DM promotes high filtration state, and resulting in increased excretion of uric acid by kidney.^[35]^

Several limitations should be considered in this study. First, we only draw conclusions based on the present cross-sectional study, it is difficult to get a cause-and-effect relationship between serum UA/Cr and MetS. Second, we do not take dietary habits into consideration as we do not have sufficient information. But dietary habits can affect serum uric acid levels. Additional studies need to be undertaken to clarify the underlying molecular mechanism that associates serum UA/Cr with MetS.

## 5 Conclusion

The present study revealed that serum UA/Cr ratio was significantly higher in MetS patients than that in controls. After the multivariate adjustments for the confounders, serum UA/Cr was still an independent risk factor for MetS.

## Acknowledgments

We thank all of the participants for their contribution to this study.

## Author contributions

**Formal analysis:** Jing Tao, Guo-Qing Li, Zhao Wang.

**Methodology:** Xin Shen, Jie Li, Lin-long He.

**Validation:** Jing Tao, Erdenbat Cha, Pei-Pei Gu.

**Writing – original draft:** Jing Tao.

**Writing – review & editing:** Jun Liu, Wei Zhu.
